# Chronic Kidney Disease As a Potential Indication for Renal Denervation

**DOI:** 10.3389/fphys.2016.00220

**Published:** 2016-06-08

**Authors:** Margreet F. Sanders, Peter J. Blankestijn

**Affiliations:** Department of Nephrology and Hypertension, University Medical Centre UtrechtUtrecht, Netherlands

**Keywords:** renal denervation, chronic kidney disease, hypertension, sympathetic nervous system, renin-angiotensin-aldosterone-system

## Abstract

Renal denervation is being used as a blood pressure lowering therapy for patients with apparent treatment resistant hypertension. However, this population does not represent a distinct disease condition in which benefit is predictable. In fact, the wide range in effectiveness of renal denervation could be a consequence of this heterogeneous pathogenesis of hypertension. Since renal denervation aims at disrupting sympathetic nerves surrounding the renal arteries, it seems obvious to focus on patients with increased afferent and/or efferent renal sympathetic nerve activity. In this review will be argued, from both a pathophysiological and a clinical point of view, that chronic kidney disease is particularly suited to renal denervation.

## Introduction

Renal denervation (RDN) is an invasive procedure in which a catheter is percutaneously introduced into the renal arteries. By applying radiofrequency energy against the blood vessel wall, nerve fibers surrounding the artery are damaged (Steigerwald et al., 2012). Sympathetic nerves fibers are the specific target (Schlaich et al., 2009a). The procedure is non-selective, meaning in this context that both afferent and efferent pathways are affected (Booth et al., 2015). So far, RDN is mostly applied to apparent treatment resistant hypertensive patients (aTRH). Resistant means uncontrolled blood pressure despite the use of ≥3 optimally dosed blood pressure lowering drugs, including a diuretic, or treatment with ≥4 blood pressure lowering drugs (Rossignol et al., 2015). When applying RDN, the assumption is that renal sympathetic nerves are too active and that this activity is the main contributor to hypertension in these patients. This assumption might actually not be true for all hypertensive patients. RDN studies so far have all shown an exceptionally large range in blood pressure lowering effect (Bhatt et al., 2014; Azizi et al., 2015). Especially the first sham-controlled trial, Symplicity HTN-3, did not show superiority of RDN to a sham procedure in lowering blood pressure and raised many questions (Bhatt et al., 2014). Secondary analyses and subsequent studies indicated that possibly in many cases the denervation was incomplete, because of too few or inappropriate location of ablation points (Kandzari et al., 2015). There was also uncertainty about medication change during the studies. These and possibly other unidentified factors may have substantially affected the outcome of the study. Furthermore, there is increasing debate on which patient categories should be offered this therapy. The major disadvantage of the aTRH patient as candidate for RDN is that this type of patients does not represent a distinct disease condition. In fact the opposite is true, with RDN being applied to very mixed groups of patients (Verloop et al., 2013; Persu et al., 2014a). So, failure to prove RDN efficacy might partly be explained by incomplete nerve ablation and partly by inaccurate patient selection. Therefore, we believe that RDN research should be focused on more distinct patient groups, in whom it is theoretically likely that they suffer from (highly) activated renal sympathetic nerves. In this review it will be argued that hypertensive patients with chronic kidney disease (CKD) could be such a target population, and indeed there is now evidence available to suggest that CKD patients may benefit from RDN (Hering et al., 2012; Kiuchi et al., 2013; Schlaich et al., 2013; Ott et al., 2015).

## Renal denervation in patients with CKD and hypertension

RDN is performed from within the renal arteries and aims at disrupting renal sympathetic nerve traffic. It is well known that (renal) sympathetic nerve activity is increased in CKD patients and that the prognostic consequences of this may be significant (Zoccali et al., 2002). Therefore it seems logical to investigate CKD as a specific indication for RDN. The arguments to support this will be discussed from both a clinical and a pathophysiological stance.

### Unmet need in CKD from a clinical point of view

Hypertension is highly prevalent in CKD patients and is both a cause and a consequence of chronic kidney failure (defined as kidney damage and/or impaired kidney function for at least 3 months, with health implications) (Rossignol et al., 2015). More than 20% of individuals with CKD suffer from hypertension, increasing to over 80% in patients with stage 4 kidney disease (Mahmoodi et al., 2012). In a recent meta-analysis the additional mortality and end-stage kidney disease (ESKD) risk of hypertension in CKD patients was investigated. They showed that the risk of (cardiovascular) mortality and ESKD was not much different in hypertensive CKD patients compared to CKD patients without hypertension (Mahmoodi et al., 2012). The hazard ratio remained the same after a sensitivity analysis in which the definition “hypertension” also included blood pressure lowering drug use. This suggests that the risk of cardiovascular mortality and ESKD is partially blood pressure independent. Several other studies have shown that in CKD patients with inadequately controlled blood pressure, outcome is particularly poor (de Nicola et al., 2011; Daugherty et al., 2012; de Beus et al., 2015; Thomas et al., 2016). Prevalence of aTRH in hypertensive CKD patients ranges from 23 to 42%, increasing with severity of kidney disease (Muntner et al., 2010; Mahmoodi et al., 2012; Tanner et al., 2013; de Beus et al., 2015). Such patients have a poorer prognosis despite extensive pharmacological treatment. Both the degree of albuminuria and reduction of GFR are associated with outcome and with the presence of aTRH (Sarnak et al., 2003; Foster et al., 2007; Tanner et al., 2013). Although often accompanied by comorbidities, such as diabetes and hypertension, CKD as such is an independent risk factor for cardiovascular disease and mortality (Mahmoodi et al., 2012; Matsushita et al., 2015). Close interaction between the heart and kidney is often called cardio-renal syndrome. Secondary cardiac involvement in CKD has been defined as chronic renocardiac syndrome type 4 (Ronco et al., 2008). Across all stages of reduced GFR, primary CKD can lead to decreased cardiac function, cardiac hypertrophy and to higher risk of cardiovascular events (Ronco et al., 2008; Hatamizadeh et al., 2013). Furthermore, as is addressed in the next paragraph, increased sympathetic nerve activity is also an independent predictor of morbidity and mortality in patients with ESKD (Zoccali et al., 2002). It seems clear that given the fact that aTRH is so common in CKD and related to poor outcome, there is a clinically relevant yet unmet need to improve the quality of treatment. This “call for action” was emphasized in a recent review (Rossignol et al., 2015). In the next section will be discussed how RDN might be an effective treatment for these high risk patients.

### Rationale from a pathophysiological point of view

The prominent role of the sympathetic nervous system (SNS) in CKD and hypertension has extensively been discussed by us and others (Schlaich et al., 2009b; Vink and Blankestijn, 2012; Grassi et al., 2015; Sata and Schlaich, 2016). We will briefly summarize this. In many studies sympathetic activity is measured by quantifying muscle sympathetic nerve activity (MSNA), which is the centrally originated sympathetic outflow toward the resistance vasculature. Organ specific sympathetic activity can be assessed by regional spillover techniques, including that to the kidneys (Grassi et al., 2015). Regardless of the underlying cause of disease, sympathetic nerve activity is increased in most patients with CKD (Blankestijn, 2004). Even in early stages of the disease, this overactivity is already present. It increases in parallel with the progression of kidney function impairment (Grassi et al., 2011). Importantly, markers of sympathetic activity, such as MSNA, noradrenaline and neuropeptide Y are on average elevated in CKD patients, but show considerable variation (Zoccali et al., 2002, 2003; Blankestijn, 2004). In 1992, Converse et al. compared sympathetic activity of haemodialysis patients with normal subjects and also with haemodialysis patients who underwent bilateral nephrectomy (Converse et al., 1992). MSNA appeared to be increased in haemodialysis patients and hypertensive patients with chronic kidney failure who are not yet on dialysis (Converse et al., 1992; Ligtenberg et al., 1999; Klein et al., 2003; Neumann et al., 2007). After bilateral nephrectomy in haemodialysis patients, MSNA decreased to normal levels, whereas no change in MSNA was observed after unilateral donor nephrectomy (Converse et al., 1992; Klein et al., 2003). So, removing a healthy kidney does not affect MSNA (despite GFR reduction), whereas removing diseased kidneys will lower MSNA. This finding suggests that the sympathetic overactivity is not explained by a reduced kidney function *per se* or by the uremic state, but that it is generated within the diseased kidneys (Hausberg et al., 2002; Klein et al., 2003). There is much evidence that kidney ischemia is the primary cause for sympathetic nerve activation. Inducing ischemia by acute renal artery stenosis in conscious rats results in both neurogenic and humoral responses (activation of the renin-angiotensin-aldosterone system, RAAS) and consequently in hypertension (Navar et al., 1998). In humans, MSNA was significantly reduced after angioplasty of renal artery stenosis (Miyajima et al., 1991). In hypertensive patients with polycystic kidney disease, a condition characterized by regional hypoxia, sympathetic nerve activity is increased, although kidney function was not impaired (Klein et al., 2001; Bernhardt et al., 2007). Sympathetic nerve stimulation from the brain to the kidney and vice versa seems to be a vicious cycle: interruption of the renal afferent nerves by dorsal rhizotomy (a definitive method for afferent RDN) prevented hypertension, secretion of noradrenaline from the posterior hypothalamic nuclei and prevented the progression of renal disease in rats (Campese et al., 1995). As earlier explained, catheter-based RDN is non-selective and thus targets both afferent and efferent renal sympathetic pathways (Booth et al., 2015; Sata and Schlaich, 2016).

## Interaction between sympathetic nerves and renin-angiotensin-aldosterone-system

As has long been recognized, the SNS and the RAAS are often simultaneously upregulated. For instance, after angioplasty in patients with renal artery stenosis, activity of both SNS and RAAS decrease. This is also illustrated by Klein et al: in patients with chronic kidney failure, both MSNA and the level of plasma renin activity were higher compared to controls and reacted parallel to each other along with changes in extracellular volume status (Klein et al., 2003). There is strong evidence that these systems also interact in their contribution to hypertension and progression of kidney failure. In the renal ablation rat model (renal mass reduction), kidney function can be preserved by ACE inhibition (ACEi), angiotensin-II receptor blocker (ARB), or by alpha- or beta blockade (Joles and Koomans, 2004). Treating hypertensive, chronic kidney failure patients with ACEi or ARB, lowers blood pressure and MSNA (Ligtenberg et al., 1999; Klein et al., 2003). Furthermore, intravenous administration of angiotensin-II to healthy individuals results in an increase in MSNA, independently of baroreceptor reflexes (Matsukawa et al., 1991). It is thought that the stimulated RAAS (by angiotensin-II) leads to hypertension in several different ways: directly by arteriolar constriction, via baroreceptor reflexes, via sympathetic nerve terminals and ganglia, via the central nervous system and via the kidneys by influencing salt and water handling (Reid, 1992). The effects on the SNS seem to play a significant role: RDN in rats attenuates hypertension that is caused by chronic angiotensin-II infusion (Hendel and Collister, 2006). Similar, Eriguchi et al. found that in rats with cardiorenal syndrome, induced by chronic L-NAME administration (causing NO depletion), bilateral RDN decreased local RAAS activity (Eriguchi et al., 2015). It is clear that interaction between the two systems is bidirectional. This could be due to bidirectional cause-effect relations, a common origin, or both.

## Lowering sympathetic nerve activity

**Figure 1:**
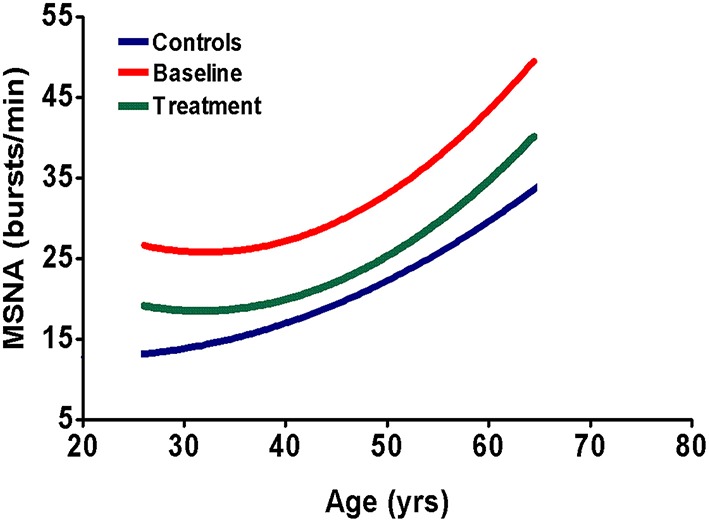
**Schematic representation of sympathetic nerve activity in CKD patients and normal subjects**. The blue line represents muscle sympathetic nerve activity (MSNA) in healthy subjects, the red line represents MSNA in untreated CKD patients and the green line represents MSNA in CKD patients, when treated chronically with a RAAS-inhibitor (Ligtenberg et al., 1999; Klein et al., 2003; Neumann et al., 2004, 2007; Siddiqi et al., 2011). Sympathetic activity increases with age, irrespective of treatment. The figure shows that chronic treatment with RAAS-inhibitors does not result in full normalization of MSNA. This indicates the need for additional sympatholytic therapy.

As stated earlier, MSNA represents the sympathetic outflow toward the resistance vasculature. It is thought to be involved in the pathogenesis of hypertension. Indeed, in an earlier study we found a positive relationship between MSNA levels and blood pressure (Siddiqi et al., 2009). In order to appreciate the effect of RDN on the SNS, it is important to know to what extent the currently often used drugs lower sympathetic activity in disease conditions characterized by sympathetic hyperactivity. It seems logical to aim at normalization. Insufficient reduction, i.e. not normalization, could suggest an unmet need. Basically, there are at least two types of sympathetic activity quantified by MSNA: a control level and an overactivated state. The control activity is generated by the central nervous system, influenced by baroreceptors and is present in every individual, even in bilaterally nephrectomized subjects (Blankestijn and Ritz, 2011). As explained, overactivity can be generated in the diseased kidney. In patients with sympathetic overactivity, the aim of therapy is to reduce this activity, back to levels comparable to normal subjects. We were the first to show that an ACE-inhibitor reduces MSNA in CKD patients (Ligtenberg et al., 1999; Neumann et al., 2007). However, MSNA did not normalize during treatment with normal dosages of ACEi or ARB (Figure 1) (Klein et al., 2003). Insufficient suppression has been confirmed in multiple clinical trials studying hypertensive CKD patients (Neumann et al., 2004, 2007; Siddiqi et al., 2011). When moxonidine (a centrally acting sympatholytic agent) was added to the treatment, both blood pressure and MSNA further decreased (Neumann et al., 2004). Monotherapy of certain oral beta-blocking agents leads to a reduction in MSNA in hypertensive and in chronic heart failure patients (Wallin et al., 1984; de Matos et al., 2004). However, like the MSNA lowering effect of RAAS inhibitors, sympathetic activity does not reach control levels: MSNA can be further decreased for example by regular exercise (Fraga et al., 2007). Carvedilol (a beta-blocker) added to standard therapy (including at least one RAAS inhibitor) in hemodialysis patients with chronic heart failure reduced morbidity and mortality, which could be due to sympatholytic actions (Azevedo et al., 2001; Cice et al., 2003). One could argue that there might be a dose-effect relation: increasing the dosage, to double or three times the normal dosage of RAAS inhibition or beta-blocking agents, would possibly further reduce MSNA. There is not much known about the dose-related sympathetic lowering effects of drugs. It is also unclear whether MSNA can completely normalize when only targeting the SNS, pharmacologically or otherwise. Since components of the RAAS and SNS are often simultaneously released, it seems reasonable to believe that a combination therapy of RAAS- and beta-blockade is essential when both systems are expected to be overactivated. Changes in activity of SNS after RDN, e.g. measured by MSNA, have not been consistently shown (Hart et al., 2013; Hering et al., 2013; Vink et al., 2014a). Evidence of parallel activation of RAAS and SNS indicate that variables of RAAS might also be indicative of an RDN effect, a hypothesis that is already supported by some early results by our group (Vink et al., 2014b). To conclude, in view of the pathophysiology, there is rationale to target both RAAS and SNS in order to adequately lower sympathetic overactivity. It is worth addressing the hypothesis that the combination of RAAS inhibitors and RDN is more effective than either intervention alone.

## Effects of RDN in CKD patients and in experimental studies

Is there any information on the effects of RDN in CKD? In RDN research, CKD patients were originally excluded from participation, mainly because of lack of safety data. Data now available seem to suggest that the procedure is safe, including in more advanced CKD, but only few studies investigated the effects of RDN specifically in a CKD population (Hering et al., 2012; Kiuchi et al., 2013, 2016; Schlaich et al., 2013; Ott et al., 2015). Longer follow-up reports describe a slight decrease in creatinine clearance, which could have many explanations, for instance, change in medication use or natural progression due to existing comorbidities (Krum et al., 2014; Tsioufis et al., 2015). In the CKD population, available studies showed a significant blood pressure reduction up to 2 years after the procedure, although most studies were limited by a small sample size, lack of control group and the uncertainty of medication adherence (Hering et al., 2012; Kiuchi et al., 2013; Schlaich et al., 2013; Ott et al., 2015; Kiuchi et al., 2016). No worsening of kidney function was reported. In fact, in some studies the opposite was found: Ott and colleagues observed beneficial effects on kidney function. In this pilot study in CKD patients with hypertension, decline of creatinine clearance slowed down after RDN (Ott et al., 2015). These potentially beneficial effects were also demonstrated in experimental studies. For example, in the study by Eriguchi et al. it became clear that both hydralazine (a direct vasodilating agent) and bilateral RDN lowered the L-NAME induced hypertension. However, the denervated rats showed protective effects, associated with local RAAS inhibition, that were blood pressure independent (Eriguchi et al., 2015). Interestingly, the results of this experimental study might be in line with the previously cited meta-analysis, showing that the morbidity and mortality risks in CKD patients are partially blood pressure independent (Siddiqi et al., 2011). So these early studies indicate that RDN in (advanced) CKD is safe. Based on limited evidence, RDN might even be renoprotective, an effect that could be blood pressure-independent.

## Perspectives

There are several aspects of the topic of this review that have not yet been touched upon here and seem appropriate to briefly discuss. First of all, we need to focus on how to improve the selection for RDN *within* the CKD population. Clinical studies investigating baseline differences between responders and non-responders to RDN did not consistently produce useful pretreatment predictors (Kaiser et al., 2014; Persu et al., 2014b; Vink et al., 2014b; Kandzari et al., 2015). In a recent review, potential strategies for selecting CKD patients for RDN are described in more detail (de Beus et al., 2014). These strategies are more based on the pathophysiology and could therefore be a potential focus of further research. Another important issue is the recent knowledge of the ability of renal sympathetic nerves to re-innervate after RDN. Although long-term follow-up studies, up to three years after RDN, showed a persistent blood pressure lowering effect, preclinical research demonstrated both functionally and anatomically that re-innervation occurred within 12 months after the procedure (Mulder et al., 2013; Esler et al., 2014; Krum et al., 2014; Booth et al., 2015). It should be investigated whether this is likely to happen in humans as well. Despite of the uncertainties of the procedure, the concept of a single intervention with prolonged effect is very attractive and most likely cost effective, especially when the price of RDN devices decreases. A so far unexplored collateral idea is the potential environmental benefit of RDN. There is emerging awareness that metabolites of pharmaceutical drugs inevitably pollute the environment (Valcarcel et al., 2013). The consequences are poorly understood but potentially disastrous (Cok et al., 2011). RDN as a one-time non-pharmaceutical treatment may be more environmentally friendly. Although the rationale behind RDN in CKD patients is strong, there is reason to be cautious. As the use of contrast imaging is part of the intervention, there has been some concern about contrast induced nephropathy in CKD patients. Most nephrologists believe that this complication is rare provided that the patient is sufficiently hydrated. It is important to realize that RDN is an elective procedure. There is sufficient time to adequately prepare the patient for the procedure and the risk seems minimal. There is concern on possible detrimental effects of the procedure on renal artery anatomy. There are some reports on renal artery abnormalities, such as stenosis, observed during follow-up (Templin et al., 2013; Persu et al., 2014c). Most of these studies lack a control group, so it is uncertain whether the stenosis was indeed caused by the intervention or a feature of the natural disease course in these high risk patients.

## Summary and conclusions

We are only at the beginning of correctly positioning RDN in the field. Up to now, RDN is mainly applied to patients with *apparent* TRH. It is now clear that this non-specific indication is not supported by knowledge on the pathophysiology of a specific condition. Nephrologists and hypertension specialists tend to think that *true* TRH is a very rare condition. In fact, aTRH seems to be a very heterogeneous population, including non-adherence, white coat hypertension, insufficiently dosed patients, high salt intake and undiagnosed secondary forms. Admittedly, aTRH is rather common, and should perhaps be redefined as “poorly controlled blood pressure for whatever reason.” Therefore, we need to change our way of thinking and use a more logical, possibly blood-pressure independent, approach for patient selection, i.e., based on the knowledge of the clinic and the pathophysiology. In CKD patients there is a clear unmet need, because aTRH is so often present, persistent and associated with poor prognosis. Furthermore, there is rather convincing evidence that the kidneys are involved in the pathogenesis and that the renal sympathetic nerves play a role. The bottom line is: in which patients will the addition of sympatholytic therapy such as RDN to existing standard therapy, that in most cases includes a RAAS inhibitor, reduce sympathetic activity. CKD is one of the disease conditions in which there is already some evidence that the addition of sympatholytic therapy to RAAS inhibition may improve prognosis. Therefore, it seems appealing to focus on this disease condition.

## Author contributions

All authors listed, have made substantial, direct and intellectual contribution to the work, and approved it for publication.

## Funding

Renal denervation related research at the Department of Nephrology and Hypertension at the University Medical Centre Utrecht is supported by grants from The Netherlands Organization for Health Research and Development (ZonMw), the Dutch Kidney Foundation and an unrestricted grant from Medtronic (Sympathy).

### Conflict of interest statement

The authors declare that the research was conducted in the absence of any commercial or financial relationships that could be construed as a potential conflict of interest.
